# Supplementary material for: Differences in diacylglycerol acyltransferases expression patterns and regulation cause distinct hepatic triglyceride deposition in fish
Source: Commun Biol. 2024 Apr 19;7:480. doi: 10.1038/s42003-024-06022-x (PMC11031565; doi:10.1038/s42003-024-06022-x)
Supplement: Supplementary file 2 — Supplementary Information [file 42003_2024_6022_MOESM2_ESM.pdf]

Supporting Information

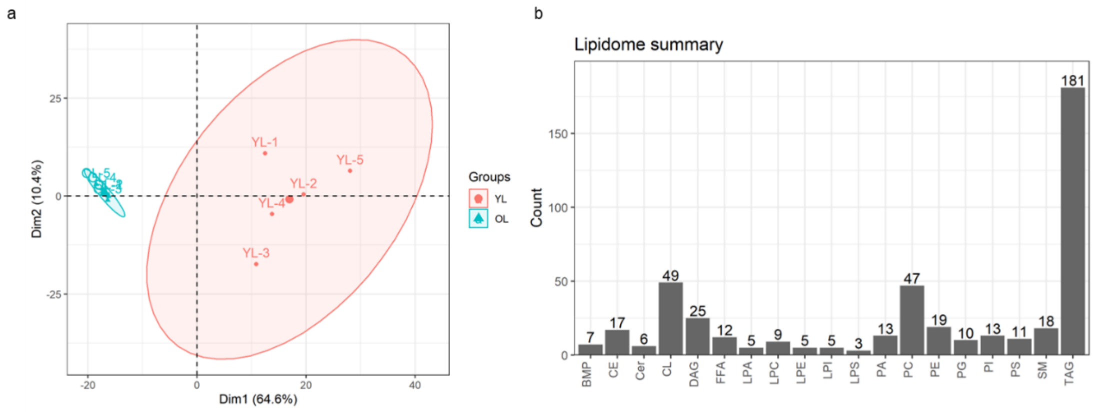

Supplementary Figure 1. Lipid analysis of the liver in large yellow croaker and rainbow trout.

a: Principal components analysis (PCA) scatter plot for hepatic lipid in large yellow croaker and rainbow trout fed same diet. YL, large yellow croaker liver (Blue); OL, rainbow trout liver (Red). b: Total lipid classes identified in hepatic large yellow croaker and rainbow trout after 10 weeks feeding trial. BMP, bis monoacylglycerol phosphate. CE, cholesterolester. Cer, Ceramide. CL, cardiolipin. DAG, diacylglycerol. FFA, free fatty acid. LPA, lysophosphatidic acid. LPC, lysophosphatidylcholine. LPE, lysophosphatidylethanolamine. LPI, lyso phosphatidylinositol. LPS, lysophosphatidylserine. PA, phosphatidic acid. PC, phosphatidylcholine. PE, phosphatidylethanolamine. PG, phosphatidylglycerol. PI, phosphatidylinositol. PS, phosphatidylserine. SM, sphingomyelin. TAG, triacylglycerol.

a

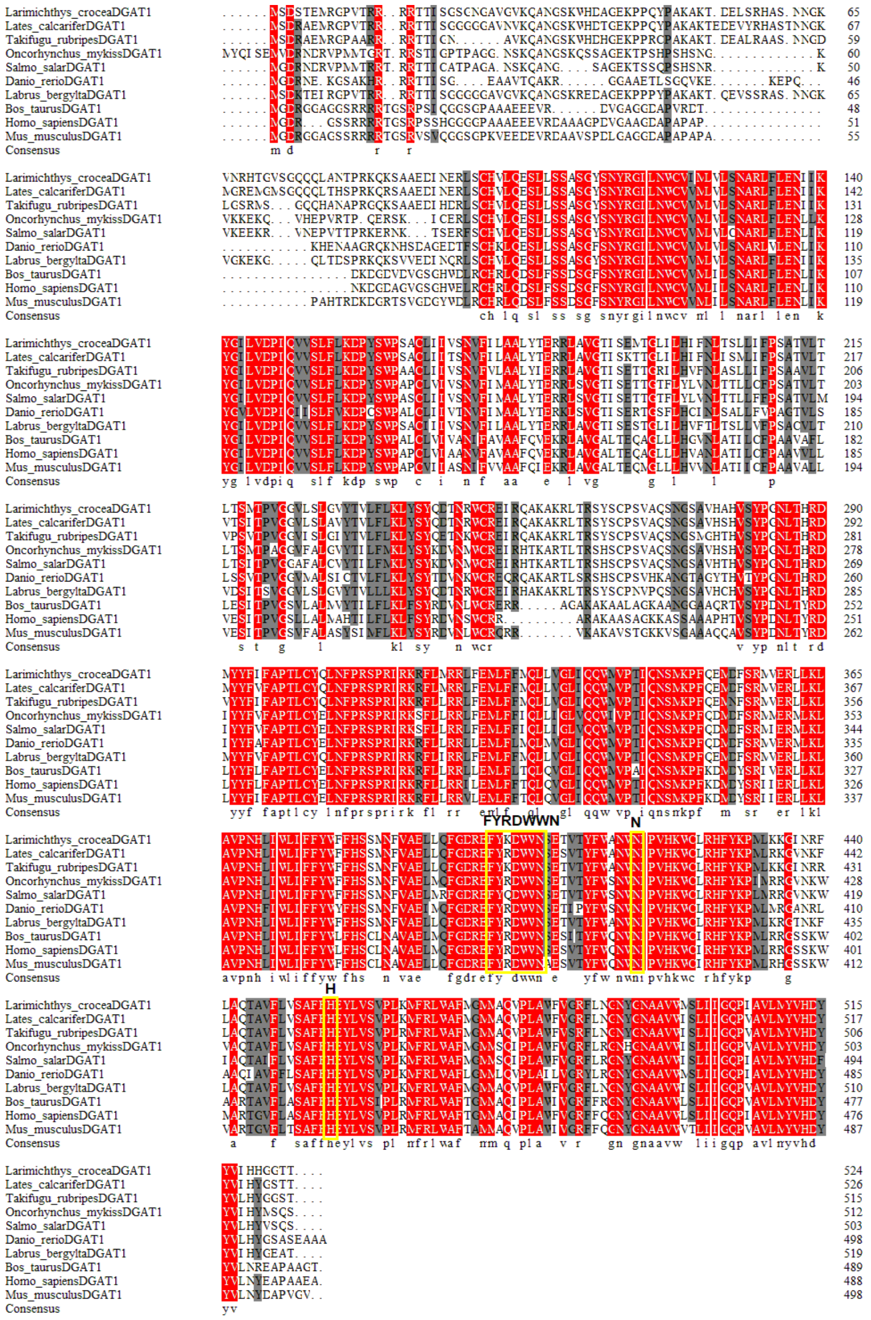
Larimichthys_croceaDGAT1
Lates_calcariferDGAT1
Takifugu_rubripesDGAT1
Oncorhynchus_mykissDGAT1
Salmo_salarDGAT1
Danio_rerioDGAT1
Labrus_bergyltaDGAT1
Bos_taurusDGAT1
Homo_sapiensDGAT1
Mus_musculusDGAT1
Consensus
FYRDWWN
N
H

b

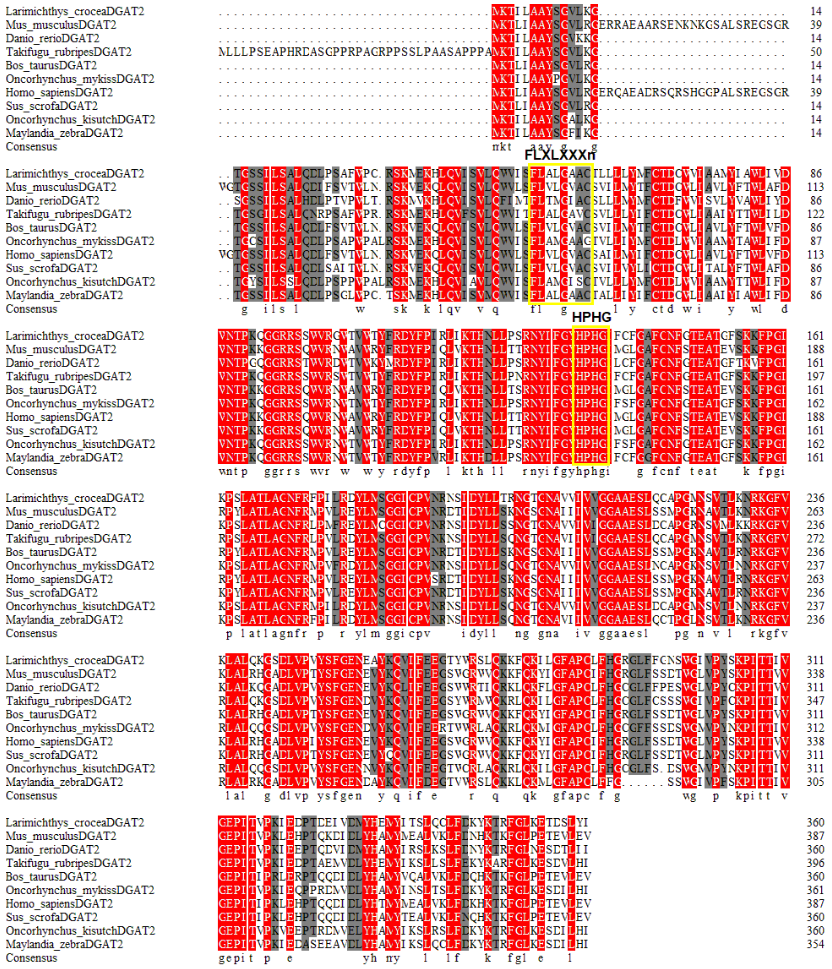

Supplementary Figure 2. Bioinformatics analysis of DGAT1 and DGAT2 in large yellow croaker and rainbow trout. Comparison of the deduced amino acid sequences of DGAT1(a) and DGAT2(b) in large yellow croaker and rainbow trout with other fish species, mammals and human. The conserved amino acid residues (FYRDWWN) and putative active sites (N and H) within the DGAT1 were highlighted in bold. Potential active site and neutral lipid binding domain residues: HPHG and FLXLXXXn residues in DGAT2 were shown in yellow box.

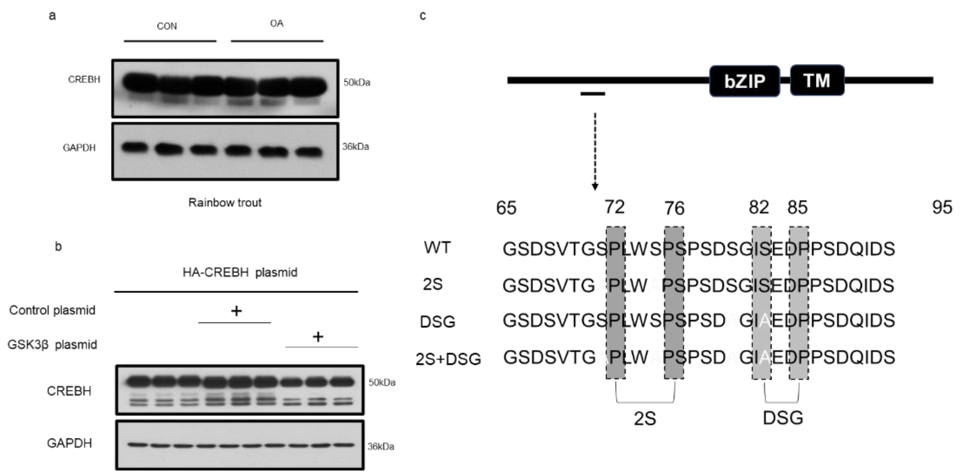

Supplementary Figure 3. Regulation of CREBH by GSK3β–mediated phosphorylation. OA incubation for 12h had no effect on the protein levels of CREBH in rainbow trout (a). Overexpression of large yellow croaker GSK3β could reduce the expression of HA-CREBH in HEK-293T cells (b). Conserved phosphorylation sites of large yellow croaker CREBH (c).

Supplementary Figure 4: scans of immunoblots in Fig. 5.

Fig. 5h

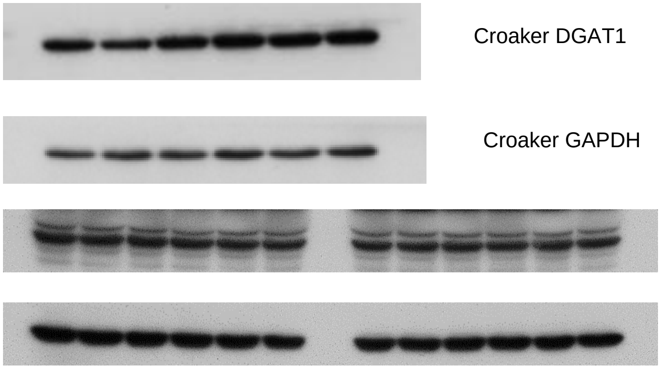

Croaker DGAT1

Croaker GAPDH

Rainbow trout DGAT1

Rainbow trout GAPDH

Supplementary Figure 5: scans of immunoblots in Fig. 8.

Fig. 8a

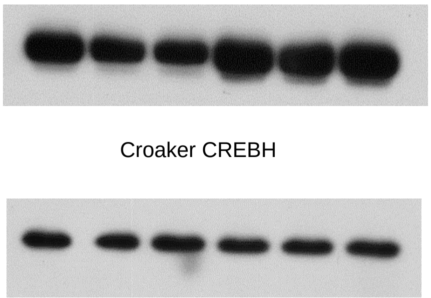

Croaker CREBH

Croaker GAPDH

Fig. 8b

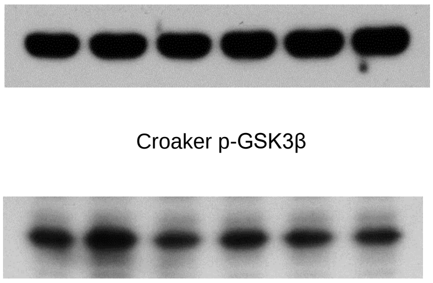

Croaker p-GSK3β

Croaker GSK3β

Fig. 8d

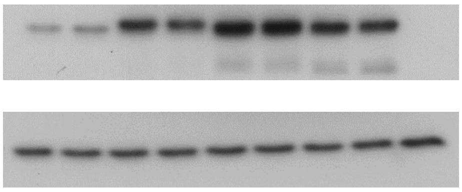

Croaker HA-CREBH

Croaker GAPDH

Supplementary Table 1. Primer sequences for DGATs gene cloning, qPCR analysis and promoter cloning.

Primer	Sequences5'-3'	Primer Information
YDGAT1-ORF-F	ATGAGCGACAGTACCGAAATGAGAG	ORF cloning
YDGAT1-ORF-R	CTATGTAGTGCCCCCATGATGAATGAC	ORF cloning
YDGAT2-ORF-F	ATGAAGACCATTCTTGCTGCATACTC	ORF cloning
YDGAT2-ORF-R	TCAGTTGATGTACAAGCTGTCGG	ORF cloning
RDGAT2-ORF-F	ATGAAGACCATACTTGCTGCCTACTCCGG	ORF cloning
RDGAT2-ORF-R	TCATTGGATGTGCAGGATGTCACTCTC	ORF cloning
RDGAT1-ORF-F	ATGTATCAAATTTCGGAAATGGTCGACAG	ORF cloning
RDGAT1-ORF-R	TCATGATTGTGACATGTAATGTATTACATAG	ORF cloning
YDGAT1-RT-F	GGTATCTTGGTGGACCCCATTCA	YDGAT1-q-PCR
YDGAT1-RT-R	TGAGCACCGTGGCTGAAGGAAAGA	YDGAT1-q-PCR
YDGAT2-RT-F	TTCGGTGCTTTCTGCAACTTCG	YDGAT2-q-PCR
YDGAT2-RT-R	AAGGATGGGGAAGCGGAAGT	YDGAT2-q-PCR
ODGAT1-RT-F	GTCTTCCTGGTCTCCGCCTTCTTT	ODGAT1-q-PCR
ODGAT1-RT-R	CCAATGATGAGCGAAATCCAGACC	ODGAT1-q-PCR
ODGAT2-RT-F	CAGTGGGTCATCTCCTTCCTCG	ODGAT2-q-PCR
ODGAT2-RT-R	TGATGAGCCTGATGGGAAAGTA	ODGAT2-q-PCR
YDGAT1-A-F	GAGTGCAGCGGAGGATATCAACG	Absolute quantification
YDGAT1-A-R	GCCTGGGTAGGAGACATGAGCG	Absolute quantification
YDGAT2-A-F	CCCCGAAGCAAGGTGGGAGG	Absolute quantification
YDGAT2-A-R	GCCCGGAGCACACTGCAGAGAC	Absolute quantification
ODGAT1-A-F	CCGGGACTGGTGGAACTCTGAGACG	Absolute quantification
ODGAT1-A-R	TCATGATTGTGACATGTAATGTATTACA	Absolute quantification
ODGAT2-A-F	ATGAAGACCATACTTGCTGCCTACTCCGG	Absolute quantification
ODGAT2-A-R	GGACTGGCATCCGGAAGTTTCCAGCC	Absolute quantification
YDGAT1-PRO-F	CCAACTTCTGCTTCCTCCCACAA	YDGAT1 promoter
YDGAT1-PRO-R	CTCAGCGGCTTCAGTCACGCAGTTTT	YDGAT1 promoter
YDGAT2-PRO-F	CCACAAGGTTCAATACTGGGTCC	YDGAT2 promoter
YDGAT2-PRO-R	GATCAGTCTGAGTGCCGCCGC	YDGAT2 promoter
ODGAT1-PRO-F	AGGTAGCCGCAAACTCTATCGC	ODGAT1 promoter
ODGAT1-PRO-R	TCAACCTTATCTTGCTGGAATTGCAC	ODGAT1 promoter
ODGAT2-PRO-F	AGAACCTTGCGGAACACCTGAG	ODGAT2 promoter
ODGAT2-PRO-R	AACTGTGGCTCCGGGAATAAACAG	ODGAT2 promoter
YDGAT1-CHIP-F	GCTCTCAACAGCTGTGCCCGAACGGATC	ChIP-PCR
YDGAT1-CHIP-R	CAGCTCGCGGTGGCGGTGCAAGTGAACG	ChIP-PCR
YDGAT1-mut-F	TGGACAGGAATGACATGCGGCTGCCATTG	Promoter mutant
YDGAT1-mut-F	GCATGTCATTCCTGTCCAATCCGAACGCG	Promoter mutant

Supplementary Table 2. Sequences of the primers for transcription factors cloning and q-PCR.

Primer	Sequence 5'-3'
pCS2+YCREBH-F	CGATTCGAATTCAAGGCCTCTCGAGATGGAACAGTTCCGAGATCAGGAC
pCS2+YCREBH-R	CTCACTATAGTTCTAGAGGCTCGAGTTACATGTCATCAGCATGCGGTCGC
pCS2+YCREBH(N)-F	CGATTCGAATTCAAGGCCTCTCGAGATGGAACAGTTCCGAGATCAGGAC
pCS2+YCREBH(N)-R	CTCACTATAGTTCTAGAGGCTCGAGTTAAGTCTGAGCTGGCTTGTTGGAT
pCS2+YCREBH(2S)-F	CgcccctctctgggccCCTTCTCCCAGTGACAGTGGG
pCS2+YCREBH(2S)-R	GggcccagagaggggcGCCGGTCACAGAATCACTTCC
pCS2+YCREBH(DSG)-F	TgacgccgggatcgccGAGGACCCTCCATCAGACCAG
pCS2+YCREBH(DSG)-R	CggcgatcccggcgtcACTGGGAGAAGGGGACCAGA
pCS2+HA-YCREBH-F	CGATTCGAATTCAAGGCCTCTCGAGATGTACCCATACGATGTTCCAGATTACGC TGAACAGTTCCGAGATCAGGACTG
pCS2+HA-YCREBH-R	CTCACTATAGTTCTAGAGGCTCGAGTTACATGTCATCAGCATGCGGTCGC
l-creb3l3-RT-F	TTTAGCAAAGGAAGGCGTGACTC
l-creb3l3-RT-R	TGCTTATTGCGAATCTTTCTGCG
pCS2+OCREBH-F	CGATTCGAATTCAAGGCCTCTCGAGATGTCAGTCACTGAGGATGTTCCT
pCS2+OCREBH-R	CTCACTATAGTTCTAGAGGCTCGAGTTACATCTCGTCCGAGCGGTGTTGC
O-creb3l3-RT-F	TGGGAACGGCTGTGGCTTAT
O-creb3l3-RT-R	TCTCGTCCGAGCGGTGTTG
l-chrebp-RT-F	AGGACCAGGGATGTGCTGAATA
l-chrebp-RT-R	AGGCGGATAAGGGTATGACTGA
l-usf1-RT-F	CAGATGGAGCTGCGGCAGTAAG
l-usf1-RT-R	GGTGCGATTGTCCTTTGATTGG
l-usf2-RT-F	AGGCTGCGTTTGCTGACCAC
l-usf2-RT-R	CCGCCGTTACTGAAAGGGTT
