# Supplementary material for: Differences in diacylglycerol acyltransferases expression patterns and regulation cause distinct hepatic triglyceride deposition in fish
Source: Commun Biol. 2024 Apr 19;7:480. doi: 10.1038/s42003-024-06022-x (PMC11031565; doi:10.1038/s42003-024-06022-x)
Supplement: Supplementary file 3 — Description of Supplementary Materials [file 42003_2024_6022_MOESM3_ESM.docx]

**Description of Additional Supplementary Files**

**File name:** Supplementary Data 1

**Description:** The source lipidomic data behind the graphs in the paper.
